# Use of a multi-drug regimen gemcitabine, 5-fluorouracil, irinotecan, cisplatin, bevacizumab, docetaxel, and cyclophosphamide (GFIP/BDC) for heavily pretreated relapsed epithelial ovarian, fallopian tube and primary peritoneal cancer

**DOI:** 10.1186/s13048-019-0506-4

**Published:** 2019-04-25

**Authors:** Samantha Cohen, Melissa Schwartz, Peter Dottino, Ann Marie Beddoe

**Affiliations:** Department of Obstetrics, Gynecology and Reproductive Sciences, Division of Gynecologic Oncology, Icahn School of Medicine at Mount Sinai, One Gustave L. Levy Place, Box 1170, New York, NY 10029 USA

## Abstract

**Background:**

Epithelial ovarian cancer has the highest fatality rate of all gynecologic malignancies. Although the majority of patients achieve complete clinical response after initial cytoreductive surgery and platinum-based chemotherapy, most recur and almost all will eventually acquire platinum-resistance for which treatment options become limited. The objective of the study was to describe response and tolerability of metronomic chemotherapy regimen GFIP/BDC, a modification of the G-FLIP regimen, in patients with persistent or recurrent epithelial ovarian, fallopian tube, and primary peritoneal cancer.

**Methods:**

A retrospective descriptive analysis of 20 patients from a single academic institution who received combination GFIP/BDC therapy from January 1, 2011 to August 31, 2016 for persistent or recurrent EOC/FT/PP. Treatment consisted of a 2-day combination of gemcitabine 300 mg, 5-fluorouracil 500 mg/m2, irinotecan 20-30 mg/m2, cisplatin 20 mg/m2, bevacizumab 4 mg/kg, docetaxel 20 mg/m2, and cyclophosphamide 200 mg/m2 administered every 14 days. Toxicities were retrospectively graded using CTCAE v4.0.

**Results:**

Twenty patients were identified with a median age 57.5 years (range 32–71). A total of 85% of patients were non-Hispanic white, 90% had cancer of high-grade serous histology, and all had a GOG performance status of 0–1. Patients had received a median of 3 prior regimens and 95% were platinum-resistant. Median number of cycles of GFIP/BDC administered was 9 (range 3–48) and patients remained on treatment for a median of 5.1 months (range 1.5–24). Eleven patients (55%) experienced a partial clinical response with a median duration of 6 months (range 1.5–20). Six patients (30%) survived progression free for at least 6 months.

Ten patients (50%) experienced at least one grade 3/4 adverse event. Grade 3 adverse events were hematologic (*n* = 5), constitutional (*n* = 3), gastrointestinal (n = 3), neurologic (*n* = 2), and vascular (*n* = 1). There was only one grade 4 adverse event which was severe neutropenia. Patients discontinued treatment due to disease progression 65% (*n* = 13), toxicity 20% (*n* = 4), patient preference 10% (*n* = 2), and 5% (n = 1) is currently on treatment.

**Conclusions:**

Selected patients with epithelial ovarian, fallopian tube or primary peritoneal cancer who have failed multiple lines of conventional cytotoxic treatment may benefit from GFIP/BDC. Toxicity might be a limiting factor for administration.

## Background

Epithelial ovarian cancer (EOC) has the highest fatality rate of all gynecologic malignancies. It is estimated that in 2019 there will be 22,530 new cases and 13,980 deaths from ovarian cancer in the United States [1]. Although the majority of patients achieve complete clinical response after initial cytoreductive surgery and platinum-based chemotherapy, approximately 80% of patients will recur [2]. Fewer than 20% of patients with advanced ovarian cancer will survive long-term [3]. Even among patients considered initially to be platinum-sensitive, almost all will eventually acquire platinum-resistance and options for treatment become limited. At this point in the disease course, overall response to alternative agents is poor and considered to range from 7 to 20% [2]. New treatment strategies are desperately needed in the setting of heavily pretreated patients, especially for those patients with acquired platinum-resistant disease. This challenging scenario in the treatment of EOC has led clinicians to evaluate potential regimens used to treat other solid tumors.

Cytotoxic chemotherapy is designed for use at the maximum tolerated dose (MTD) and is typically administered at defined intervals. Planned treatment breaks such as 2–3 weeks allow for patient recovery from harmful side effects such as bone marrow suppression [4, 5]. Although these regimens are often initially effective, debilitating side effects at the MTD, long-term sequela related to toxicity, and ultimate failure of sustained treatment response has led investigators to explore alternative drug delivery strategies [6]. A different treatment approach using metronomic administration of chemotherapy has thus emerged in recent years as an alternative. This treatment modality utilizes either frequent or continuous drug delivery without extended breaks [6].

Ovarian and other cancers have a high rate of relapse and ultimately these tumors become drug resistant [7]. Metronomic chemotherapy which has moved from a preclinical phase to clinical trials, addresses drug resistance in cancer by shifting the target from the tumor cell to the tumor vasculature. Instead the purpose is to inhibit the host endothelial cell of the tumor’s neovasculature. This antiangiogenic approach avoids myleosuppression and other dose-limiting side effects that would necessitate treatment breaks, thus allowing damage of the slower proliferating tumor endothelial cells [7–9]. Furthermore, there is evidence to suggest that some of these dosing/scheduling approaches work best when combining chemotherapeutics, and efficacy can be intensified when an antiangiogenic drug is added to the regimen [5].

This metronomic approach to treatment led clinicians to develop a novel protocol in the treatment of pancreatic cancer which similar to ovarian cancer, is characterized by a high rate of relapse and ultimate drug resistance. Kozuch et al. described a novel treatment strategy for metastatic pancreatic cancer, whereby irinotecan was added to gemcitabine, leucovorin, cisplatin and 5-fluorouracil (G-FLIP) in 34 patients [10]. The authors discuss the rationale behind this combined regimen including the synergistic interactions that have been described between the different drugs. This includes the antiproliferative effect of irinotecan and 5-FU at low fixed doses when irinotecan proceeds 5-FU. It also takes advantage of the increased cytotoxic effects of cisplatin by formation of DNA adducts when given 24 h after gemcitabine infusion [10–13]. Additionally it has been shown that 5-FU potentiates the antitumor effect of cisplatin by inhibiting platinum-DNA adduct repair and maximum synergistic effect occurs when 5-FU proceeds cisplatin [10, 14]. The authors also discuss possible mechanisms by which irinotecan may overcome cisplatin resistance. These include inhibiting DNA replication/repair complexes from working further then platinum adducts and restoring platinum efflux mechanisms [10].

In this cohort, the majority of patients were heavily pretreated or had failed gemcitabine or a combination of gemcitabine, 5-fluorouracil (5-FU) and cisplatin (GFP) in the past. Dosing was based on previous trials that had evaluated these drugs as doublet therapy and drugs were administered over 48 h and given every 2 weeks. Eight patients (24%) had a partial response and 7 (21%) had disease stabilization. The regimen was well tolerated and the majority of grade 3–4 toxicities were hematologic and manageable [10].

Results from further phase II investigations of the G-FLIP regimen continued to demonstrate clinical activity, with Goel et al. reporting a disease control rate of 68% for 21 patients [15]. Clinical benefit rate (CBR) was 52%. This is noteworthy when compared to the CBR of gemcitabine alone, which the authors comment is considered to be 23.8%. When combined with earlier phase I data, the median number of cycles was 7, grade 3–4 toxicity was low, and thrombosis rate was 12.5% and consistent with the baseline expected incidence in this population. The authors concluded that this is a feasible regimen with high response rate and acceptable toxicity.

Further study of G-FLIP included the addition of bevacizumab to this protocol. Bruckner et al. reported on the use of the G-FLIP regimen in combination with low dose bevacizumab for the treatment of refractory pancreatic cancer with demonstrated benefit [16]. The authors theorized that as a metronomic approach, G-FLIP produces a high rate of stable disease and regression of tumors that is often slow but prolonged, and there may be enhanced efficacy with the addition of an angiogenesis inhibitor. In general, the use of bevacizumab is of particular value in the treatment of platinum-resistant ovarian cancer and its efficacy has been demonstrated in a phase III trial [17]. In the treatment of relapsed ovarian cancer, metronomic dosing with the addition of an antiangiogenic inhibitor to maximize its effect, was therefore the premise behind the clinical use of this protocol in practice.

As stated above, administering lower doses of chemotherapy at more frequent intervals may assist in overcoming resistance and therefore deliver a substantially lower cumulative dose of cytotoxic therapy [18–20]. This metronomic approach to delivery of chemotherapy shortens the time between cycles and may prevent effective recovery of damaged cancer cells [6]. In the setting of recurrent ovarian cancer, metronomic-based treatments have been studied in the laboratory as well as reported clinically and have demonstrated notable efficacy [21, 22]. One phase II clinical trial studied metronomic oral cyclophosphamide in combination with bevacizumab (given weekly for the first 3 weeks and then every 2 weeks thereafter) in a cohort of 70 patients [23]. Partial response was achieved in 17 patients (24%) and stable disease in 44 patients (63%). As the investigators of this landmark clinical trial describe, angiogenesis is regulated by redundant pathways and therefore combination therapy potentially maximizes antiangiogensis efficacy [23]. We therefore sought to describe response and tolerability of GFIP/BDC, a modification of the metronomic delivered G-FLIP regimen [10, 15, 16, 24], in patients with heavily pretreated persistent or recurrent epithelial ovarian, fallopian tube, and primary peritoneal carcinoma (EOC/FTC/PPC). This regimen, similar to the G-FLIP protocol, represents a combination therapy that includes an antiangiogenic agent.

## Methods

### Patients

We retrospectively examined patients who received salvage combination GFIP/BDC therapy from January 1, 2011 to August 31, 2016. All patients had histologically confirmed EOC/FTC/PPC and were heavily pretreated and refractory to conventional treatment. Institutional Review Board approval at the Icahn School of Medicine at Mount Sinai (ISMMS) was granted for this study. A total of 20 patients were identified from a single academic institution (ISMMS). All patients had a GOG performance status of 0 or 1.

### Treatment

The GFIP/BDC regimen consisted of a 2-day combination of gemcitabine, 5-FU, irinotecan, cisplatin, bevacizumab, docetaxel, and cyclophosphamide. Treatment was administered intravenously in an outpatient setting every 2 weeks. Day 1 consisted of gemcitabine 300 mg in 250 cc normal saline over 30 min, 5-fluorouracil 500 mg/m2 given over 24 h, irinotecan 20-30 mg/m2 in 50 cc normal saline over 90 min, and bevacizumab 4 mg/kg in 100 cc normal saline over 1 h. Day 2 consisted of cisplatin 20 mg/m2 in 250 cc normal saline over 30 min, docetaxel 20 mg/m2 over 1 h, and cyclophosphamide 200 mg/m2 over 30 min. One cycle was 14-days. Cycles were repeated until disease recurrence or progression or unacceptable toxicity.

### Response and toxicity criteria

While receiving GFIP/BDC, patients were surveilled with serial office visits, physical exams, and computerized tomography imaging. Serum CA-125 levels were monitored before every cycle. Disease response was defined as follows: 1) complete response—disappearance of all previously evaluable lesions; partial response—any clinically relevant decrease in size of evaluable lesions, ≥75% decrease in serum CA-125 level over three samples or normalization of CA-125 level [16]; progressive disease—one or more new lesions identified on imaging or unequivocal progression; stable disease—neither partial response or progression of disease.

Toxicities were retrospectively graded using the National Cancer Institute Common Terminology Criteria for Adverse Events Version 4.0 (CTCAE v4.0). In the event of grade 3 hematologic adverse events, appropriate supportive therapies were administered and either the entire regimen was stopped or selected cytotoxic agents were discontinued from subsequent cycles. GFIP/BDC treatment was stopped for any grade 4 adverse event.

## Results

From January 2011 to August 2016, 20 patients with heavily pretreated recurrent or progressive epithelial ovarian or peritoneal carcinoma were treated with GFIP/BDC. The median age of the patients was 57.5 years (range 32 to 71). 85% of patients were non-Hispanic white, while the remaining 15% were of Asian. Five patients (15%) had a BRCA mutation. Patient characteristics with regards to primary site of malignancy, histologic subtype, stage and grade of malignancy at diagnosis, and primary debulking surgery are summarized in Table 1. The majority of patients had cancer of serous histology (90%) and advanced-stage disease at time of diagnosis (95%). All patients in this study had a GOG performance status of 0 to 1.

**Table 1:** Patient characteristics

Characteristic	No. of Patients	Percentage (%)
Primary Site of Malignancy		
Ovary	17	85
Fallopian Tube	1	5
Peritoneal	2	10
Histologic Type		
Papillary serous	18	90
Endometriod	1	5
Carcinosarcoma	1	5
Histologic Grade		
G2	3	15
G3	17	85
Stage at Initial Diagnosis		
IA	1	5
IIIA	1	5
IIIB	1	5
IIIC	13	65
IV	4	20
Primary Cytoreductive Surgery		
No gross residual	4	20
Optimal	9	45
Suboptimal	1	5
Unknown	6	30

Prior to GFIP/BDC, all patients had received at least two lines of chemotherapy (range 2 to 17). Patients received a median of 3 prior lines, 2 lines—6 patients, 3 lines—5 patients, 4 lines—3 patients, 5 lines—2 lines, and > 5 lines—4 patients, before being given this multi-drug regimen (Table 2). All patients were treated with at least one platinum-based combination chemotherapy regimen prior to starting the GFIP/BDC regimen, median 2 (range 1 to 6). At time of GFIP/BDC administration, 95% of patients were platinum-resistant.

**Table 2:** Number of previous chemotherapy lines

No. of Regimens per Patient	No. of Patients	Percentage (%)
Any Regimen		
2	6	30
3	5	25
4	3	15
5	2	10
>5	4	20
Platinum-Based Combination Chemotherapy		
1	3	15
2	8	40
3	5	25
4	0	0
5	3	15
>5	1	5

The rationale behind initiation of the GFIP/BDC regimen was abstracted from clinical charts and reported as persistence in 4 patients, progression in 11 patients, and recurrence in 4 patients. The median number of GFIP/BDC cycles administered was 9 (range 3 to 48) and patients remained on treatment for a median of 5.1 months (range 1.5 to 24). The most common reason that the GFIP/BDC regimen was discontinued was due to progression of disease on treatment which occurred in 11 patients (55%). Patients also discontinued treatment due to recurrence 5% (1 patient), persistent disease 10% (2 patients), patient preference 10% (2 patients), and unacceptable adverse side effects 20% (4 patients). One patient (5%) is currently still receiving treatment.

Disease response to GFIP/BDC is summarized in Table 3. Overall, eighteen patients (90%) experienced a clinical response or stabilization of disease with BFIP/BDC (Table 3). Eleven patients (55%) had a partial response and 7 patients (35%) had stable disease. The median duration of response was 5 months (range 1.5 to 20). Eight patients (45%) survived progression free for at least 6 months. One patient with platinum-resistant disease had a partial response to GFIP/BDC, which translated as a normalization of CA-125 level and decrease in size of liver lesions on imaging, and had a progression-free survival of 20 months.

**Table 3:** Response to GFIP/BDC

Response	No. of Patients	Percentage	Median Duration of Response (range)
Partial	11	55%	6 months (1.5 – 20)
Stable	7	35%	4.5 (2 – 6)
Progressive	2	10%	N/A

Eighteen patients (90%) had at least one adverse event from GFIP/BDC. Two patients (10%) had no reported adverse effects from the multidrug regimen. Toxicities were largely hematologic or constitutional (Tables 4 and 5). Hematologic adverse events ranged from mild anemia to febrile neutropenia requiring intensive care unit hospitalization and affected thirteen patients (65%). The most common hematologic toxicity was neutropenia. All constitutional adverse events were related to degrees of fatigue. Thirteen patients (65%) experienced some level of fatigue, of which three patients (15%) had severe fatigue that was debilitating and limited self-care.

**Table 4:** Number of toxicities associated with GFIP/BDC

Toxicity	None	Grade 1	Grade 2	Grade 3	Grade 4
Hematologic	3	1	11	4	1
Constitutional	7	2	8	2	0
Gastrointestinal	14	0	3	2	0
Nutrition	18	0	1	1	0
Neurologic	18	1	1	2	0
Pain	19	0	1	0	0
Vascular	19	0	0	1	0
General	19	0	1	0	0

**Table 5:** Hematologic toxicity associated with GFIP/BDC by type and grade

Toxicity	None	Grade 1	Grade 2	Grade 3	Grade 4
Anemia	0	1	1	1	0
Neutropenia	0	0	7	1	1
Thrombocytopenia	0	0	4	2	0

Ten patients (50%) experienced at least one grade 3 or 4 adverse event with three patients experiencing more than one grade 3 or 4 adverse events due to GFIP/BDC. Most of these toxicities were hematologic. Grade 3 hematologic adverse events were anemia (1 event), thrombocytopenia (2 events), and neutropenia (2 events). No bleeding episodes were reported. Packed red blood cell transfusion was given to two patients. All patients with grade 3 neutropenia were treated with granulocyte colony-stimulating factor. One of these patients had neutropenic sepsis requiring hospitalization and no additional cycles of GFIP/BDC were given to this patient. The remaining grade 3 adverse events were gastrointestinal (2 events), nutrition-related (1 event), neurologic (2 events), and vascular (1 event). The gastrointestinal-related events included two small bowel obstructions and one patient with severe nausea and vomiting requiring hospitalization and total parenteral nutrition (TPN). Another patient also needed to be admitted to the hospital for TPN due to extreme weight loss and inability to tolerate nutrition by mouth. The neurologic events were unexplained hallucinations in one patient and a syncopal episode in the other. The vascular event was a severe hypertensive episode requiring hospitalization and intravenous medication. Lastly, the sole grade 4 adverse event was a case of severe neutropenia.

## Discussion

Administration of traditional cytotoxic chemotherapy is the delivery of treatment in single doses or short courses at the highest dose possible without prohibitive levels of toxicity which is referred to as the MTD [5]. Most of these drugs are designed to inhibit or kill as many tumor cells as possible and administration at the MTD requires prolonged treatment breaks [5, 6]. MTD-based therapy results in acute side effects such as hair loss, nausea and vomiting and myelosuppression. This can lead to diminished quality of life, result in significant treatment delays, and necessitate supportive care drugs that add to financial toxicity of treatment and are associated with other side effects. Long-term sequela of treatment such as cardiac, neurologic and reproductive outcomes can be further disabling and moreover, resistance to cytotoxic chemotherapy leads to ultimate failures in treatment [5, 6].

The rationale behind metronomic chemotherapy includes the use of a lower dose then the MTD and dosing frequency is characterized by either a continuous or frequent administration [4, 6]. Unlike conventional treatment with a rise and fall in plasma concentration, steady plasma concentration of the drug is a feature of metronomic therapy, and thus is associated with few side effects and less need for supportive care therapy [4]. In contrast to targeting proliferating cells, the aim of treatment is to damage tumor-associated endothelial cells and disrupt angiogenesis [4, 6, 8]. The treatment breaks that are necessary in traditional regimens based on the MTD are felt to allow these endothelial cells to recover by use of the intact p-53 repair mechanism, thus limiting the antiangiogenic effect of treatment [9]. In contrast, the schedule of drug administration that characterizes the metronomic approach, exploits the slower proliferation of tumor endothelial cells whereby exposing them to damage and limiting their ability to recover [9]. Other mechanisms characteristic of metronomic therapy are thought to include selective inhibition of migration of the endothelial cell, increase in thrombospondin 1 (TSP1) an endogenous inhibitor of angiogenesis and a decrease in bone marrow-derived endothelial progenitor cells [4, 6]. In addition, endothelial cells, due to their relative genetic stability are less likely to become drug resistant as are tumor cells [4]. Moreover, the strength of metronomic chemotherapy can be enhanced when given in combination with specific agents that target angiogenesis such as antibodies against vascular endothelial growth factor (VEGF) or VEGF receptor 2 [4, 6, 16]. Other antiangiogenic agents in combination with metronomic chemotherapy have shown promise in preclinical models [9].

Another feature with this approach is the use of combination therapy that targets different aspects of a tumor’s potential vulnerability [6]. Furthermore, most tumors that are exposed to traditional chemotherapy regimens development drug resistance, either intrinsic and/or acquired [5]. Already mentioned is the idea that treatment breaks during conventional chemotherapy allow the endothelial cells of the vasculature to recover. These endothelial cells are considered genetically stable relative to the genetically diverse cancer cell. This results in the limited antiangiogenic effect typically seen with conventional chemotherapy. As Kerbel et al. explains, with administration of metronomic-based chemotherapy, the damage to these vascular support cells, can potentially overcome the drug resistance by attacking the tumor through a “side effect” antiangiogenic approach [5].Namely, tumor cells that are chemoresistant to a particular drug, may respond to that same drug via this side effect mechanism when the supporting vasculature is damaged. In their review, the authors site outcome data from the treatment of non-small-cell lung cancer as well as other solid tumors, whereby patients considered resistant to a particular chemotherapeutic agent were retreated with the same drug administered metronomically with improved clinical response [5, 25]. Furthermore, the goal of treatment in what is typically a heavily pretreated metastatic disease population, is disease stabilization with a focus on symptom control and quality of life. In general, the goal of antiangiogenic therapy is to interrupt new vessel formation and disrupt the tumor vasculature. As opposed to eliminating existing disease that is the goal of conventional chemotherapy, antiangiogenic therapy induces long-term change and is meant to be continuous with stabilization of disease as the intended clinical outcome [26].

Other cytotoxic drug combinations utilizing a metronomic approach to chemotherapy have shown activity in overcoming tumor resistance in the treatment of ovarian cancer [22, 23]. To our knowledge, this is the first report in the literature evaluating the efficacy and toxicity of GFIP/BDC for heavily pretreated ovarian cancer patients. In addition, the durability of response to this regimen has never been reported. This series demonstrates that selected EOC/FTC/PPC patients who have failed multiple lines of conventional cytotoxic treatment may benefit from GFIP/BDC. In our small cohort of patients, despite the fact that 95% had platinum-resistant disease, 90% had a partial response (11 patients) or disease stabilization (7 patients) on the GFIP/BDC regimen. Median duration of response was 6 months, ranging from 1.5 to 20 months. Three patients (15%) achieved disease response that lasted over 6 months.

Although, a majority of patients responded to GFIP/BDC or had stabilization of disease, toxicity could be a limiting factor to administration. Information regarding toxicities was abstracted retrospectively from patient charts and therefore some toxicities may have been underestimated or the grade misclassified. Most toxicities were hematologic (neutropenia, thrombocytopenia, anemia), constitutional (fatigue) or gastrointestinal (nausea, diarrhea, ileus or small bowel obstruction) in nature. Grade 3 and 4 toxicities were largely hematologic. Hematologic toxicities of neutropenia (45%), thrombocytopenia (30%), and anemia (15%) were consistent with the toxicities seen with the G-FLIP regimen used for patients with metastatic pancreatic cancer [10, 15, 24]. A total of 65% of patients experienced fatigue with 15% having debilitating symptoms, which is again consistent with previous reports from use of a similar regimen. One patient elected to stop treatment as a result.

As this was a retrospective descriptive study with a small, heterogeneous sample size, limitations should be noted. Patients selected to receive the GFIP/BDC regimen all had a GOG performance status of 0 or 1 and therefore were a select group of patients. Further, all patients in this study were cared for by three physicians, which may have added to selection bias. Lastly, response and toxicities to the GFIP/BDC regimen were determined retrospectively and based on information abstracted from medical records. Several patients deemed to have had a partial response to treatment were based on a drop in CA-125 levels without imaging to confirm response. Toxicities could have been under reported in the medical records.

The efficacy demonstrated with GFIP/BDC regimen in our cohort is nevertheless is promising and warrants investigation in a larger subset of patients. In the treatment of relapsed platinum-resistant ovarian cancer more diverse treatment options are needed as currently there are limited options. These women carry a poor prognosis with expected low response rates to subsequent lines of therapy [2]. Frequent continuous administration of low dose chemotherapy has been shown to be more efficacious and with less toxicity in select situations [27]. The use of metronomic cytotoxic regimen may help overcome platinum-resistance and enhance the efficacy of each individual agent in a synergistic fashion [21, 22]. In addition, the regimen described here is in line with other evidence that suggests enhanced efficacy of bevacizumab when given in a metronomic fashion with other cytotoxic agents [16, 20, 23]. In addition to bevacizumab, other antianiogenic agents have also been used in this fashion. Pazopanib, an oral antiangiogenesis inhibitor, in combination with metronomic cyclophosphamide showed a median PFS of 8.4 months in patients with recurrent platinum-resistant ovarian cancer in a recent phase I study [28].

In an era of personalized medicine, a GFIP/BDC metronomic approach has demonstrated potential. Thus, salvage chemotherapy with the GFIP/BDC regimen in heavily pretreated patients with recurrent ovarian, fallopian tube or primary peritoneal carcinoma may benefit select patients. GFIP/BDC has a relatively high response rate. However, hematologic toxicities may be a limiting factor for administration and some patients may not be able to tolerate therapy without significant constitutional symptoms, namely fatigue. The evolution of this regimen in the future may also include combination with other targeted therapeutics and immunotherapeutic agents.
